# Genome-wide digital transcript analysis of putative fruitlet abscission related genes regulated by ethephon in litchi

**DOI:** 10.3389/fpls.2015.00502

**Published:** 2015-07-07

**Authors:** Caiqin Li, Yan Wang, Peiyuan Ying, Wuqiang Ma, Jianguo Li

**Affiliations:** ^1^State Key Laboratory for Conservation and Utilization of Subtropical Agro-Bioresources, China Litchi Research Center, South China Agricultural UniversityGuangzhou, China; ^2^Physiological Laboratory for South China Fruits, College of Horticulture, South China Agricultural UniversityGuangzhou, China; ^3^Bioinformation Department, Beijing Genomics Institute at ShenzhenShenzhen, China

**Keywords:** *Litchi chinensis* Sonn., fruitlet abscission, digital transcript abundance, ethephon, gene

## Abstract

The high level of physiological fruitlet abscission in litchi (*Litchi chinensis* Sonn.) causes severe yield loss. Cell separation occurs at the fruit abscission zone (FAZ) and can be triggered by ethylene. However, a deep knowledge of the molecular events occurring in the FAZ is still unknown. Here, genome-wide digital transcript abundance (DTA) analysis of putative fruit abscission related genes regulated by ethephon in litchi were studied. More than 81 million high quality reads from seven ethephon treated and untreated control libraries were obtained by high-throughput sequencing. Through DTA profile analysis in combination with Gene Ontology and KEGG pathway enrichment analyses, a total of 2730 statistically significant candidate genes were involved in the ethephon-promoted litchi fruitlet abscission. Of these, there were 1867 early-responsive genes whose expressions were up- or down-regulated from 0 to 1 d after treatment. The most affected genes included those related to ethylene biosynthesis and signaling, auxin transport and signaling, transcription factors (TFs), protein ubiquitination, ROS response, calcium signal transduction, and cell wall modification. These genes could be clustered into four groups and 13 subgroups according to their similar expression patterns. qRT-PCR displayed the expression pattern of 41 selected candidate genes, which proved the accuracy of our DTA data. Ethephon treatment significantly increased fruit abscission and ethylene production of fruitlet. The possible molecular events to control the ethephon-promoted litchi fruitlet abscission were prompted out. The increased ethylene evolution in fruitlet would suppress the synthesis and polar transport of auxin and trigger abscission signaling. To the best of our knowledge, it is the first time to monitor the gene expression profile occurring in the FAZ-enriched pedicel during litchi fruit abscission induced by ethephon on the genome-wide level. This study will contribute to a better understanding for the molecular regulatory mechanism of fruit abscission in litchi.

## Introduction

Fruit abscission, occurring during fruit development, is characterized through a high coordination of biochemical events that take place in a group of specialized cells located between the pedicel and fruitlet, known as abscission zones (AZs, Bonghi et al., 2000; Sun et al., 2009). In agricultural production, shedding of fruit is a major limiting factor of yield. Over the last few decades, it is widely believed that abscission involves in multiple changes in cell structure, metabolism and gene expression, and divides into four major steps (Patterson, 2001; Estornell et al., 2013): (i) the ontogeny of AZ, (ii) the acquisition of competences to respond to abscission signals, (iii) the onset of the cell separation, (iv) the differentiation of a protective layer. It is well achieved that plant hormones are deeply involved in abscission, and ethylene operates as an efficient accelerator for organ abscission. Although there is no clear and sufficient evidence for a direct link between the ethylene perception and the onset of abscission, it is well supported that the development of this process is concomitant with an increase in the production of ethylene (Zhu et al., 2008). In fact, application of ethephon, an ethylene-releasing compound, effectively hastens the abscission of fruit in apple (Yuan, 2007; Kolarič et al., 2011), sweet orange (John-Karuppiah and Burns, 2010), sweet cherry (Smith and Whiting, 2010), and olive (Zahra, 2014). However, aminoethoxyvinylglycine (AVG), an inhibitor of ethylene biosynthesis, blocked the fruit abscission promoted by auxins in apple (Zhu et al., 2008), while 1-Methylcyclopropene (1-MCP), an inhibitor of ethylene perception, did not affect the abscission-promoted effect of ethephon in orange (John-Karuppiah and Burns, 2010). In this regard, understanding the regulatory effects of ethylene on abscission is important for the fruit industry.

Up to date, gene expression and enzymatic studies on organ abscission have shown that ethylene either facilitates the efficacy of ethylene signaling pathways (Li and Yuan, 2008; John-Karuppiah and Burns, 2010), or activates the synthesis and secretion of several cell wall and middle lamella hydrolytic enzymes associated with the separation of cells at the AZ, such as cellulase (Abeles and Leather, 1971; MacDonald et al., 2011) and polygalacturonase (Taylor et al., 1993). A transcriptome analysis could be one of the most powerful tools to understand complicated transcriptional regulation during plant organ shedding. In order to provide a new insight into the molecular basis of ethylene-mediated abscission, there are few cases of transcriptome analyses performed using ethylene-treated pedicels as materials. In the study of ethylene-promoted citrus leaf abscission, Agustí et al. (2008, 2009) discovered the preferential accumulation gene families in laminar AZ after ethylene treatment, such as cell wall modification, lipid transport, protein biosynthesis and degradation, transcription factors (TFs), stress and pathogen-related genes and some special genes involved in signaling events. In tomato, Wang et al. (2013) compared the transcriptome difference between the AZ and neighboring portion (the basal and apical) of pedicel in a time course after ethylene treatment, proposing a possible regulatory scheme involving in tomato flower abscission. However, the comparative analysis of the transcriptome profiles involved in ethylene-promoted fruit abscission using AZ as materials is lacking, although it has long been observed that application of exogenous ethylene accelerates fruit abscission.

Litchi (*Litchi chinensis* Sonn.), an important economic fruit crop in subtropical area, has been challenged by massive fruit drop, one of the major factors causing a low yield (Yuan and Huang, 1988; Mitra et al., 2003). For example, a medium size tree may produce about 60,000 female flowers but, typically, less than 5% of flowers develop into mature fruits (Stern et al., 1995). Yuan and Huang (1988) reported that there were three to four waves of physiological fruit drop throughout fruit development in 70~90 days depending on cultivars. Wave I, wave II, and wave III of abscission occurred around 1 week, 3 weeks, and 6–7 weeks after full bloom, respectively, but wave IV was specific to cultivars with aborted seeds and occurred 2–3 weeks before harvest. Previously, few studies focus on the molecular regulation mechanism of litchi fruit abscission. Through the application of ethephon, ethylene has been proved to have an unequivocal promotive effect on litchi fruitlet abscission and increase the expression of *LcPG1* encoding a pectin-degrading enzyme (Peng et al., 2013). On the other hand, there was circumstantial evidence that there had a higher fruit abscission rate and *ACO* (1-aminocyclopropane-1-carboxylic acid oxidase) gene expression level in fruits treated by NAA (naphthalene acetic) spraying, suggesting a potential role in fruit abscission (Wu et al., 2013). Nevertheless, the comprehensive transcriptome-wide expression profiling analysis under ethylene-induced abscission has not yet been documented in litchi.

In this experiment, we performed a genome-wide digital transcript analysis on fruit abscission zone (FAZ) enriched pedicel at 0, 1, 2, 3 d time points of ethephon treatment. Our results showed that a total of 6167 ethylene-regulated genes were preferentially expressed in ethephon-treated FAZ-enriched tissues. Among them, 2730 candidate genes were considered to be involved in ethylene-promoted fruit abscission process by further Gene Ontology (GO) and KEGG pathway enrichment analyses. It was demonstrated that a range of functional categories such as plant hormone synthesis and signaling, carbohydrate metabolism, TFs and cell wall modification, were highly regulated by ethylene. These results will provide a new insight of the ethylene regulatory fruit abscission molecular mechanism in litchi.

## Materials and methods

### Plant materials and treatment

Nine 9-year-old litchi trees (*L. chinensis* Sonn. cv. Feizixiao) were randomly selected in an orchard located at South China Agricultural University in 2012 (Guangzhou, China), and blocked into three biological replicates of three trees each. At 25 d after anthesis, 20 fruit-bearing shoots (about 5~8 mm in diameter) located in different directions from each tree were tagged. Ten of them were dipped in 250 mg L^−1^ethephon solution (containing 0.05% Tween-80 surfactant) for 1 min, while the remaining 10 shoots dipped in water were used as control. Three out of ten treated shoots were used to monitor fruit abscission dynamic and the others were used for sampling. Samples were conducted at 0, 1, 2, and 3 d after treatment. Fruitlet and FAZ-enriched pedicels were collected immediately after the samples were taken back to laboratory on ice. FAZ-enriched pedicels were excised by cutting around 2 mm at each side of the abscission fracture plane (Supplementary Figure 1). After separation, all tissues were quickly frozen in liquid nitrogen and stored at −80°C for future analysis.

### Determination of fruit abscission and ethylene production rate of fruit

Cumulative fruit abscission rate (CFAR) was calculated according to our previous method (Kuang et al., 2012). Ethylene production was measured according to the method described by Yan et al. (2011) with some modifications. Two fruit from each treatment on each tree were collected and enclosed in a 30 mL airtight syringe equipped with a rubber piston for 2 h at 25°C. Air within the syringe was forced into an airtight container filled with saturated salt water with a needled inserted to allow replacement. After all the samples were collected, 1 mL air sample was then withdrawn from the headspace of the container with a syringe and injected into a GC-17A gas chromatograph (Shimadzu, Kyoto, Japan) fitted with a flame ionization detector and an activated alumina column (200 cm × 0.3 cm). The injector temperature was 120°C; the column temperature was kept at 60°C and the detector temperature at 60°C. Helium was used as carrier gas at a flow rate of 30 mL min^−1^. The ethylene production rate was expressed as microliters of C_2_H_4_ kg^−1^ h^−1^.

### Digital transcript abundance library preparation and illumina sequencing

Total RNA from FAZ-enriched pedicel was isolated using Column Plant RNAout 2.0 kit (TIANDZ, Inc, China). The quantity and quality of RNA samples were evaluated using 2100 Bioanalyzer (Agilent Technologies, Santa Clara, CA, USA). Equal amounts of total RNA from three biological replicates were pooled to construct seven libraries named CK0, CK1, CK2, CK3, ETH1, ETH2, and ETH3. For example, CK1 and ETH1 were the libraries from pedicels harvested at 1 d after water and ETH treatment, respectively. After RNA extraction, mRNA purification by Oligo (dT), fragmentation, cDNA synthesis by random hexamer primers, size selection and PCR amplification were performed by BGI-Shenzhen as described previously (Li et al., 2013).

### Data analysis for digital transcript abundance profiles

High-quality reads used for further downstream processing were filtered through the standard Illumina pipeline to remove the low-quality reads and those containing adaptor/primer contaminations. All clean reads were mapped back to the litchi genome (http://litchidb.genomics.cn, unpublished) using SOAPaligner (Version 2.21) allowing up to two nucleotide mismatches with the parameters of “-m 0 -x 1000 -s 28 -l 32 -v 2 -r 2,” which are specified on http://soap.genomics.org.cn/soapaligner.html. Clean reads mapped to reference, from multiple genes, were filtered and unambiguous clean reads were remained. For gene expression analysis, the number of unambiguous clean reads for each gene was calculated and normalized to RPKM (Reads Per Kilo base per Million reads) (Mortazavi et al., 2008). Six paired-libraries including CK0 vs. CK1, CK0 vs. CK2, CK0 vs. CK3, CK1 vs. ETH1, CK2 vs. ETH2, and CK3 vs. ETH3 were used to analyze the differential gene expression, according to the method described in Audic and Claverie (1997). Two filter criteria were used to identify differentially expressed genes (DEGs): a four-fold change in transcript levels and a FDR (False Discovery Rate) value ≤ 0.001 among every comparison.

In order to eliminate the control background noise, take day 1 treatment data for example, we excluded the DEGs identified in CK0 vs. CK1 library from the DEGs identified in CK1 vs. ETH1 library, and the obtained data was recorded as ETH1/CK1. The other two time points were done as the same way, and the result was recorded as ETH2/CK2 and ETH3/CK3, respectively. Then the union of DEGs among ETH1/CK1, ETH2/CK2, and ETH3/CK3 was defined as ethephon-responsive genes. Finally, all ethephon-responsive genes were mapped to terms in GO and KEGG databases for functional and pathway-enrichment analysis. And those genes significantly enriched in GO term analysis (*FDR* ≤ 0.05) or enriched in KEGG pathway (*Q*-value ≤ 0.05) were screened to be the candidate genes involved in the fruit abscission process. Heat maps showing expression profiles were generated using the MultiExperiment Viewer (MeV, v4.9).

### Quantitative real-time PCR

To validate the accuracy of our DTA profiles results, 41 randomly selected DEGs were evaluated by quantitative real-time PCR (qRT-PCR) after the ethephon treatment in litchi FAZ. The RNA samples for DTA analysis were also used for qRT-PCR. Gene-specific primer sequences were designed using Primer Premier 5.0 and listed in Supplementary Data Excel File 1. Purified total RNA (2 μg) from each sample was reverse-transcribed to synthesize cDNA by ReverTra Ace qPCR RT Master Mix with gDNA Remover (TOYOBO). Then, the cDNA was amplified using SYBR Green-PCR master kit (THUNDERBIRD SYBR qPCR Mix, TOYOBO) and LightCycler 480_II Real-Time PCR System (Roche). The PCR amplifications included the following condition: 95°C for 1 min, followed by 40 cycles of 95°C for 5 s, 55°C for 30 s and 72°C for 30 s. Dissociation curves were run to determine the specificity of the amplification reactions. The data were normalized using cycle threshold (Ct) value corresponding to two litchi reference genes, *EF-1α* and *GAPDH* (Zhong et al., 2011). The relative expressive level of the target genes were calculated using the ΔΔCt method. Duplicates from three biological replicates were used.

## Results

### Changes in fruit abscission rate and ethylene production

**Figure 1:**
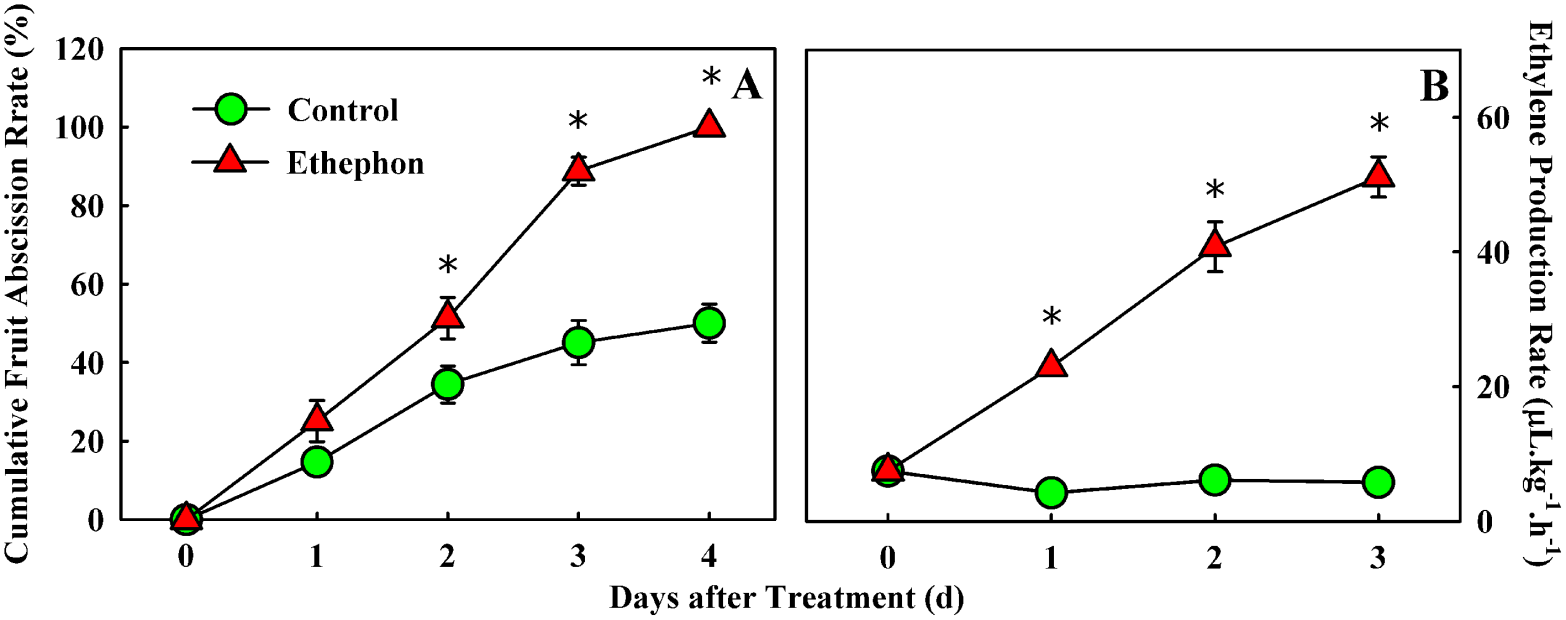
**Effect of ethephon treatment on fruitlet abscission (A) and ethylene production (B) in litchi**. Each value represented the means of three biological replicates from nine different trees, with the standard error (SE) indicated by vertical bars. Significant differences at 0.05 level are indicated with asterisk (^*^) according to *t*-test.

CFAR and ethylene production in fruitlet were compared between the control and ethephon (ETH) treatment (Figure 1). The CFARs showed similar trends (Figure 1A), which gradually increased in the first day and had no visible difference. Two days after treatment, the CFAR in ETH-treated fruitlet was significantly higher than that in the control. Consequently, 100% of the fruitlet abscised by 4 d after ETH treatment, compared with a ~50% loss in the control, indicating that ETH treatment significantly accelerated fruitlet drop. In addition, a clear impact on ethylene production was also observed in ETH-treated fruitlet. Within 3 days of observation, ethylene production in the control fruitlet remained more or less flat and kept below 8 μl kg^−1^ h^−1^. While ethylene production in the ETH-treated fruitlet increased rapidly and continuously, and achieved nearly a ten-fold higher level at day 3 than the control (Figure 1B). The increase in ethylene production suggested that ethephon application probably accelerates the fruit drop following the induction of ethylene production in fruitlet.

### Digital transcript abundance profile analysis

To explore the transcriptional changes of litchi FAZ-enriched pedicel in response to ethephon treatment, seven digital transcript abundance (DTA) tag profiles of the control (CK0, CK1, CK2, and CK3) and ETH-treated samples (ETH1, ETH2, and ETH3) were sequenced (Table 1). After quality filtering, nearly 82 million clean reads were generated from the above libraries (10–12 million reads for each library). The tag sequences of the seven libraries were mapped to the litchi genome, and 87.11, 86.93, 86.69, 87.20, 87.53, 87.37, and 86.52% of all clean reads were obtained, respectively. These results showed that both the throughput and sequencing quality were high enough for further analysis.

**Table 1:** **Statistics of digital transcript abundance library sequencing and read mapping**.

**Libraries**	**Total reads**	Total mapped reads^*^	**Unique match reads**
CK0	12,009,135	10,461,248 (87.11%)	4,491,205 (37.40%)
CK1	11,530,205	10,022,661 (86.93%)	4,400,232 (38.16%)
CK2	10,568,766	9,161,789 (86.69%)	4,003,146 (37.88%)
CK3	10,862,567	9,472,399 (87.20%)	4,147,267 (38.18%)
ETH1	12,477,700	10,921,142 (87.53%)	4,756,944 (38.12%)
ETH2	11,905,084	10,401,413 (87.37%)	4,516,857 (37.94%)
ETH3	12,284,279	10,628,560 (86.52%)	4,693,781 (38.21%)

* *Number and percentage of reads mapped onto litchi genome (unpublished data). CK and ETH mean the untreated control and ethephon treatment, the numbers following the CK and ETH are the days after treatment*.

### Confirmation of gene expression patterns by qRT-PCR

**Figure 2:**
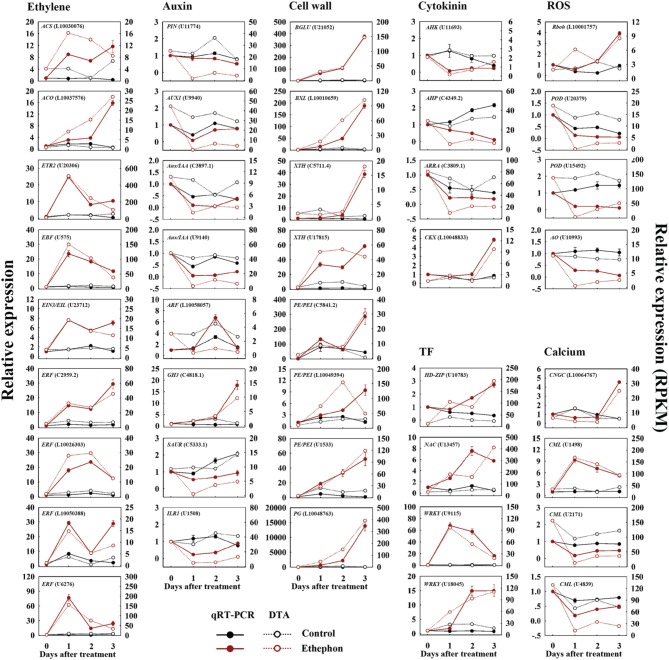
**Confirmation of candidate gene-expression pattern in FAZ-enriched pedicel during ethephon-induced fruit abscission**. qRT-PCR analysis (filled circles, left y-axis) of 41 selected genes at 0, 1, 2, 3 d after ethephon treatment. Relative expression values were normalized to the pre-treatment (0 d) expression value taken as 1. The data represent the mean values (±SE) of duplicate experiments from three independent biological samples. Broken lines (open circle, right y-axis) show expression profiling of genes in the FAZ-enriched pedicel from the DTA data, and indicate the total read count in RPKM for each gene after normalization across the seven samples: CK0, CK1, CK2, CK3, ETH1, ETH2, and ETH3. CK and ETH mean the untreated control and ethephon treatment, the numbers following the CK and ETH are the days after treatment.

Forty-one randomly selected genes were analyzed by quantitative real-time PCR to verify their expression patterns (Figure 2). Among these, transcripts encoding the key enzymes involved in ethylene biosynthesis and signaling, such as *ACS* (L10030076), *ACO* (L10037576), *ETR2* (U20306), *EBF* (U575), *EIN3/EIL* (U23712) and *ERF* (L10026303, L10050388, C2959.2, and U6276), were sharply increased at 1 d after treatment, which were consistent with the results analyzed by DTA (Figure 2). Similarly, we verified the increased expression of genes encoding those enzymes related to cell wall degradation, TFs, cytokinin metabolism and ROS production, such as *BGLU* (U21052), *BXL* (L10010659), *XTH* (C5711.4 and U17815), *PE/PEI* (C5841.2, L10049394, and U1533), *PG* (L10048763), *HD-ZIP* (U10783), *NAC* (U13457), *WRKY* (U18045 and U9115), *CKX* (L10048833) and *Rboh* (L10001757), as well as the down-regulated genes involved in auxin transport and signaling, cytokinin signaling, ROS scavenging and calcium signaling, such as *PIN* (U11774), *AUX1* (U9940), *Aux/IAA* (C3897.1 and U9140), *SAUR* (C5333.1), *ILR1* (U1508), *AHK* (U11693), *AHP* (C4349.2), *ARRA* (C3809.1), *POD* (U20379 and U15492), *AO* (U10993), and *CML* (U2171 and U4839). The expression level of one auxin early responsive gene (*GH3*, C4818.1) and one calcium signaling gene (*CML*, U1498) were increased by ETH treatment, which was identical to the DTA results. One calcium transport gene (*CNGC*, L10064767) showed down-regulated at 1 d after treatment and increased at 3 d after treatment (Figure 2). However, the expression level of one gene encoding auxin response factor (*ARF*, L10058057) was contrasted to the DTA result (Figure 2). Thus, except one *ARF* gene, all the other 40 genes showed similar expression patterns as detected by the DTA, indicating that the DTA analysis results were effective.

### Transcirptome responses during fruit abscission

After comparing three paired-libraries (CK1 vs. ETH1, CK2 vs. ETH2, and CK3 vs. ETH3), a total of 6167 genes were found to be significantly up- or down-regulated which named as ethephon-responsive genes (Supplementary Data Excel File 2). Based on GO enrichment analysis, 3249 genes (53%) were divided into the three principal GO organization categories (Supplementary Figure 2A): biological process (2437 genes), cellular component (1459 genes) and molecular function (2825 genes). The enriched GO terms included carbohydrate metabolic process, photosynthesis, extracellular region, cell wall, thylakoid, catalytic activity and oxygen binding. KEGG pathway enrichment analyses showed that a total of 3420 genes were assigned to the 118 related pathways, and the top 20 enriched pathways including plant hormone signal transduction (ko04075) and starch and sucrose metabolism (ko00500) were illustrated in Supplementary Figure 2B.

### Analysis of the candidate genes involved in the fruit abscission process

A total of 2471 and 2344 genes were selected in GO (*FDR* ≤ 0.05) and KEGG enrichment analyses (*Q*-value ≤ 0.05) with a significant level, respectively. After eliminating duplicated, 2730 genes were identified as the candidate genes involved in the fruit abscission process regulated by ethephon (Supplementary Data Excel File 3). The up- and down-regulated genes accounted for 37.44% (1022 genes) and 62.56% (1708 genes) of them, respectively.

**Figure 3:**
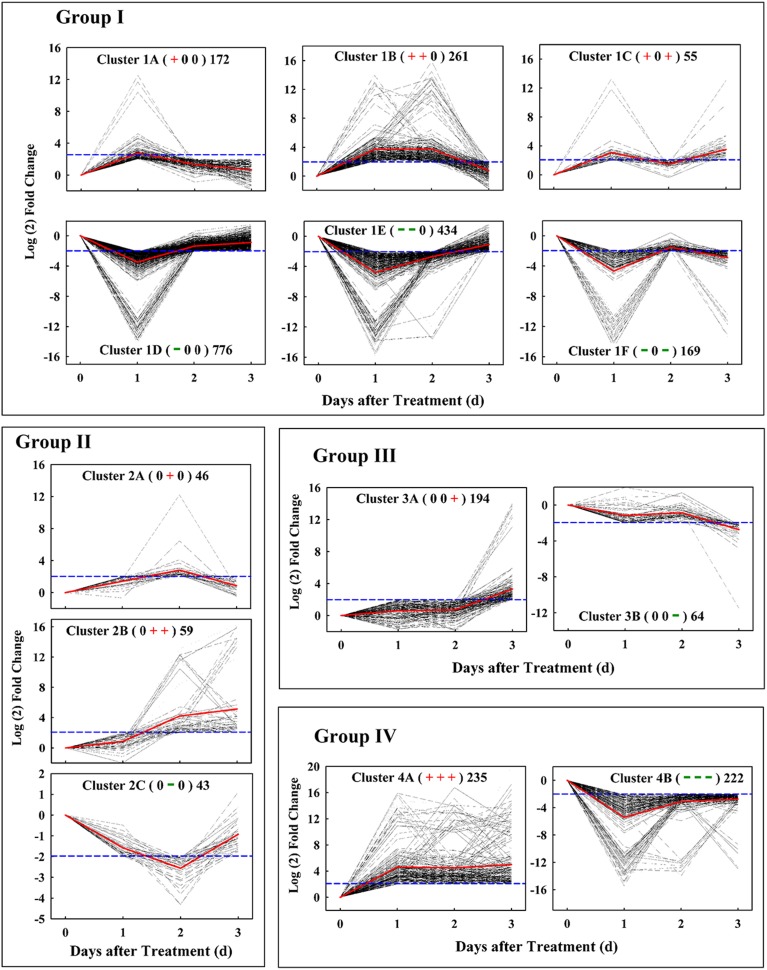
**Ethephon-responsive genes expression pattern obtained by kinetics-based clustering analysis. Group I**, cluster of genes with early and transient changes; **group II**, clusters of genes modified in their expression until 2 d after treatment; **group III**, cluster of genes with expression kinetics exhibiting late changes; **group IV**, cluster of genes with persistent changes during the whole abscission process. The + and − signs in bracket represent up- and down-regulated of genes, respectively, while 0 represents no change. The numbers on the right of bracket indicate the total numbers of genes in each cluster. All of these changes were based on a four-fold change criterion (log2 ratio) indicated by blue dotted line. Gray dotted line indicates the gene-expression levels and the average values of gene-expression level in clusters is shown by the red solid lines.

Based on their patterns of expression, these candidate genes could be classified into four groups, which consisted of genes with similar temporal patterns of expression kinetics (Figure 3). Group I included 1867 early-responsive genes whose expression were up- or down-regulated early at 1 d after treatment; Group II had 148 middle-responsive genes whose expression were not induced or suppressed until 2 d after treatment; Group III contained 258 late-responsive genes that were not regulated until 3 d after treatment; Group IV consisted of 457 constant-responsive genes that up- or down-regulated early and whose expression was maintained constant during the treatment. By hierarchical cluster analysis, each group could be subsequently divided into two to six clusters, for example, Group I included cluster 1A, 1B, 1C, 1D, 1E, and 1F which had 172, 261, 55, 776, 434, and 169 genes, respectively. In total, 723 up-regulated and 1601 down-regulated genes were found at 1 d after treatment, and 299 up-regulated and 107 down-regulated genes were found at 2 d or 3 d after treatment. These results showed that the majority (85.13%) of those candidate genes made a quick response to the ETH treatment in 24 h when no significant difference on fruit abscission rate was found between the control and the ETH treatment.

Except 101 genes encoding proteins of unknown functions, the other 2629 genes had unambiguous annotations. Those genes related to plant hormones, cell wall metabolism TFs, carbohydrate metabolism, ROS and calcium signaling were further analyzed as follows.

#### Genes related to plant hormone biosynthesis and signaling pathway

**Figure 4:**
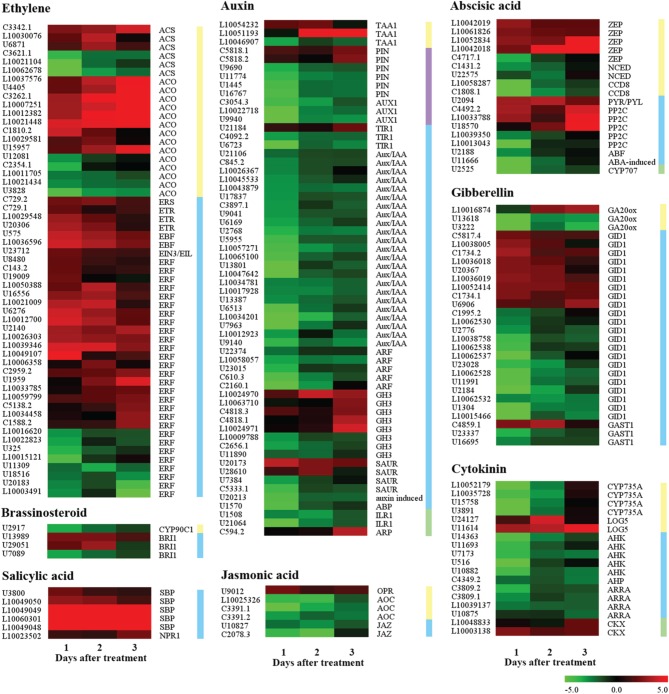
**Expression profiling of genes related to plant hormone biosynthesis, signaling, and metabolism in the FAZ-enriched pedicel after ethylene treatment**. Yellow, blue, purple, and green vertical lines indicate biosynthesis, signaling, transport and degradation genes, respectively. Gene expression levels are indicated with color bars. Additional information was presented in Supplementary Data Excel File 4.

A total of 195 candidate genes were found related to plant hormone biosynthesis and signaling pathway (Figure 4, Supplementary Data Excel File 4). Of these, 124, 16, 17, and 38 genes belonged to the Group I, Group II, Group III, and Group IV, respectively. Sixty and fifty-five genes were related to auxin and ethylene, among them, 47 auxin-related genes were down-regulated and 39 ethylene-related genes were up-regulated. These genes should be closely associated with fruitlet abscission, including those encoding auxin efflux carrier component (PIN), auxin influx carrier (AUX1), AUX/IAA protein, auxin response factor (ARF), SAUR family protein, GH3 protein, 1-aminocyclopropane-1-carboxylate oxidase (ACO), 1-aminocyclopropane-1-carboxylate synthase (ACS), AP2/ERF transcription factor and ethylene receptor (ETR), et cetera. Twenty-eight gibberellins (GA) related and 18 cytokinin-related genes were found, most of them were down-regulated, such as genes encoding gibberellin 20 oxidase (GA20ox), gibberellin receptor GID1, cytokinin hydroxylase (CYP735A), histidine kinase (AHK) and two-component response regulator (AARA), etc… Eight of eighteen abscisic acid (ABA) related genes were up-regulated including those encoding zeaxanthin epoxidase (ZEP), abscisic acid receptor (PYR/PYL) and protein phosphatase 2C (PP2C), and the repressed genes mainly encoded 9-cis-epoxycarotenoid dioxygenase (NCED), carotenoid cleavage dioxygenase (CCD), and abscisic acid 8′-hydroxylase (CYP707). Moreover, six salicylic acid (SA) related genes encoded with salicylic acid-binding protein (SBP) and regulatory protein NPR1 were highly up-regulated, and five jasmonic acid (JA) related genes mainly encoded allene oxide cyclise (AOC) and jasmonate ZIM domain-containing protein (JAZ) were decreased. These results suggested that seven classes of plant hormones were involved in the process of fruitlet abscission induced by the ETH treatment. The most important hormones were ethylene and auxin, followed by GA, cytokinin and ABA. JA and SA ranked the last according to the number of DEGs.

#### Genes encoding for transcription factors

**Figure 5:**
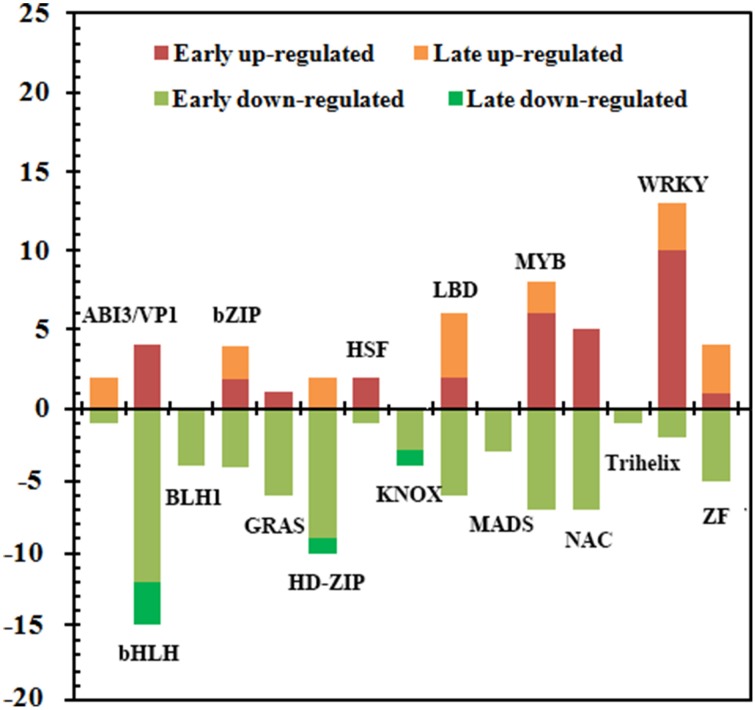
**Summary of the number of significant changes in transcription factors between the different families of ETH-responsive genes**. Changes in the abundance of 127 TFs belonging to 15 families were identified in the FAZ-enriched pedicel of litchi. Comparison of early (Group I and Group IV) and late (Group II and Group III) stages for significantly up-regulated and down-regulated transcripts revealed differences in the families of TFs during ethephon-induced abscission. Additional information was presented in Supplementary Data Excel File 6.

Except for 58 genes putatively encoding TFs related to plant hormones, there were 127 candidate TFs belonged to diverse families including *ABI3/VP1*, *bHLH*, *BLH1*, *bZIP*, *GRAS*, *HD-ZIP*, *HSF*, *KNOX*, *LBD*, *MADS-box*, *MYB*, *NAC*, *WRKY*, *Trihelix*, and *zinc finger* (Figure 5, Supplementary Data Excel File 5). Of these, 51 and 76 genes were up- and down-regulated, respectively. And 78, 9, 14, and 26 belonged to Group I, Group II, Group III and Group IV, respectively. Remarkably, 71 out of 114 TFs in Group I and Group IV were down-regulated, indicating that most TFs were repressed at 1 d after the ETH treatment. There were more than 10 members in those families including *bHLH*, *MYB*, *WRKY*, *NAC*, *LBD*, and *HD-ZIP*. Most members of *bHLH* and *HD-ZIP* families were down-regulated, while 13 of 15 members in the family of WRKY were up-regulated, and all members in the families of *BLH1*, *MADS-box*, *KNOX*, and *Trihelix* were down-regulated. Moreover, 33 of the 51 up-regulated TFs were induced at 1 d after treatment, and the largest TF family was the *WRKY* (10 genes), followed by *MYB* (6 genes) and *NAC* (5 genes), implying that those genes from these TF families could be related with triggering the transcriptional chain reaction during ETH-induced abscission.

#### Genes related to cell wall biosynthesis, degradation, loosening, and modification

**Figure 6:**
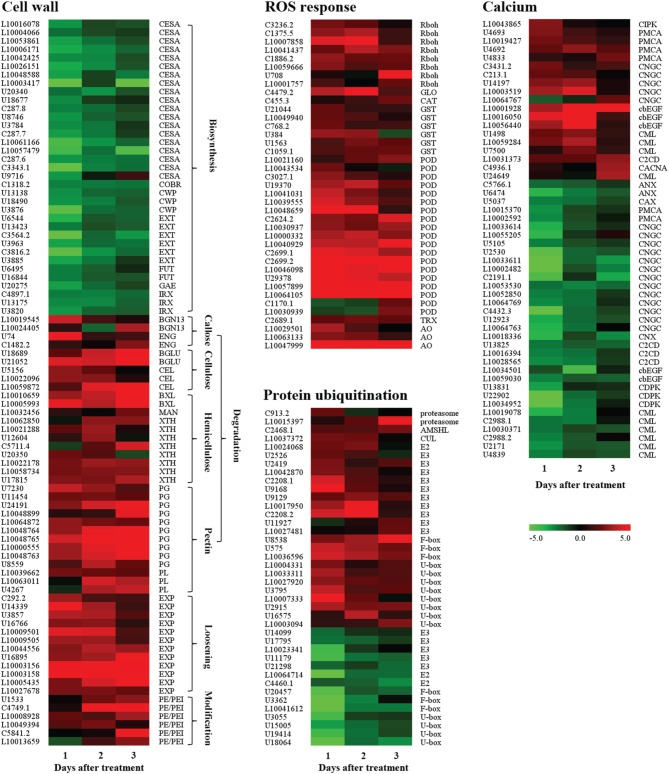
**Expression profiling of genes related to cell wall modification, ROS response, calcium signaling transduction, and protein ubiquitination in the FAZ-enriched pedicel after ethylene treatment**. Up-regulated genes involved in cell wall degradation, cell wall loosening and ROS response, and down-regulated genes related to cell wall biosynthesis were showed. All genes involved in calcium signaling transduction and protein ubiquitination were exhibited. Gene expression levels were indicated with color bars. Additional information was presented in Supplementary Data Excel File 4.

A total of 208 genes including 56, 104, 21, and 27 genes related to cell wall biosynthesis, degradation, loosing and modification were found, respectively (Supplementary Data Excel File 6). Of these, 72 and 136 genes were up- and down-regulated, respectively. Of the up-regulated genes, there were 51 cell wall degradation, loosing and modification related transcripts (Figure 6). Among them, 4 genes were related to callose degradation, like endo-1,3-β-glucosidases (*ENGs*) and β-1,3-glucanases (*BGN13s*); 5 genes were involved in cellulose degradation, such as endo-1,4-β-D-glucanases (*CELs*) and β-glucosidases (*BGLUs*); 11 genes like endo-1,4-β-mannosidase (*MAN*), xyloglucan endotransglucosylase/hydrolases (*XTHs*) and β-D-xylosidases (*BXLs*) were related to hemicellulose degradation; 13 genes such as polygalacturonases (*PGs*) and pectate lyase (*PLs*) were associated with pectin degradation; 12 expansins (*EXPs*) related to cell wall loosening and 6 pectinesterase/pectinesterase inhibitors (*PE/PEIs*) genes were involved in cell wall modification. Of the down-regulated genes, there were 35 cell wall biosynthesis-related genes (Figure 6), including those encoding cellulose synthase, extensin, glycine-rich cell wall structural protein, UDP-glucuronate 4-epimerase and xyloglucan glycosyltransferase. There were not surprised that those genes encoding enzymes mentioned above may be involved in the process of fruitlet abscission induced by ethephon. However, 101 down-regulated genes related to cell wall degradation, loosening and modification, and 21 up-regulated genes related to cell wall biosynthesis were also found in our study.

#### Genes related to photosynthesis, carbohydrate, and energy metabolism

The expression of over 93% (103 out of 110 genes) genes involved in photosynthesis pathway were strongly decreased, especially repressed exclusively at 1 day after the ETH treatment, and most of them belonged to Group I (Supplementary Data Excel File 7). The affected genes function in chlorophyll biosynthesis, thylakoid formation, chlorophyll a/b binding protein, light harvesting (PSI and PSII), electron transport and carbon fixation. These results suggested repressed expression of those encoding for chloroplast and light harvesting associated proteins by ethephon might lead to the process of chloroplast malfunction and photosynthesis inhibition.

Not surprisingly, the down-regulated photosynthesis related genes are linked with changes in the expression of genes in carbohydrate metabolism. A total of 137 candidate genes were found in this section (58 up-regulated and 79 down-regulated, Supplementary Data Excel File 7). Overall, 24 out of 32 genes involved in glycolysis and all members of the TCA cycle (7 genes), such as alcohol dehydrogenase, glyceraldehyde 3-phosphate dehydrogenase and ATP-citrate synthase, were strongly down-regulated within Group I, indicating that the degradation of sugar was inhibited during the early ETH-induced abscission. However, an increased expression of seven genes associated with fructose and mannose degradation and four genes involved in galactose hydrolysis were also found during the late induction of abscission. These results suggested that sugar degradation was inhibited during the early induction but increased during the late induction. Moreover, our data also showed that 13 out of 17 genes encoding ATPase were repressed after the ETH treatment, especially down-regulated exclusively at 1 day post-treatment (Supplementary Data Excel File 7). These results indicated that ethephon might lead to the interdiction of ATP synthesis and the high energy supply.

#### Genes related to ROS response, calcium signal transduction, and protein ubiquitination

A total of 88 transcripts belonging to the reactive oxygen species (ROS) response were preferentially regulated in the ETH-treated FAZ-enriched pedicels (Supplementary Data Excel File 8). Of these, 39 genes were up-regulated and the other 49 genes were down-regulated. Among the up-regulated genes (Figure 6), we found eight respiratory burst oxidase homolog proteins (*Rboh*) and one peroxisomal-(S)-2-hydroxy-acid oxidase (*GLO*) involved in ROS production, indicating a high ROS content might be produced in FAZ. Not surprisingly, 27 genes putatively encoding ROS scavenging enzymes of diverse families showed increased expression, and the most abundant transcripts encoding glutathione S-transferase (*GST*) and peroxidase (*POD*). However, the down-regulated ROS scavenging genes were also found, indicating that a competing activity occurred in the regulation of fruit abscission. In addition, 20 genes encoding the key enzymes related to ascorbate synthesis and metabolism, including L-ascorbate oxidase (AO) and inositol oxygenase (MIOX), showed an immediate decreased regulation at the 1 day post-treatment.

As an important signaling molecular, 52 transcripts related to calcium transport and perception displayed altered changes (Figure 6, Supplementary Data Excel File 8). Among them, 19 and 33 genes were up- and down-regulated respectively, and 46 of them belonged to Group I, indicating that most genes had instantaneous response to the ETH treatment. For example, two calcium influx transporters, *CNGC* (13 out of 18 genes) and *ANX* (2 genes), showed strongly decreased expression during the early induction. In contrast, 4 out of 6 transcripts encoding calcium efflux carrier proteins (PMCAs) were increased. These results indicated that a role regulating calcium influx and maintaining calcium level in the FAZ may be associated with the beginning of abscission process. Moreover, two calcium responsive genes, *CML* (6 out of 10 genes) and *CDPK* (3 genes), were also repressed during the early induction.

A total of 40 genes involved in protein ubiquitination were found (Figure 6, Supplementary Data Excel File 8), and 32 of them belonged to Group I and IV, indicating that most genes had a quick response to the ETH treatment. Among them, 26 genes were up-regulated, including those encoding 26S proteasome, cullin, E3 ubiquitin-protein ligase, EIN3-binding f-box protein, U-box domain-containing protein and ubiquitin-conjugating enzyme E2. However, 14 down-regulated genes involved in protein ubiquitination were also found.

## Discussion

Ethephon (2-chloroethylphosphonic acid) is a synthetic plant growth regulator discovered several decades ago, which acts by releasing ethylene when it penetrates plant tissues (Royer et al., 2006). The application of exogenous ethylene inducing fruit abscission has been observed in apple (Yuan, 2007; Kolarič et al., 2011), sweet orange (John-Karuppiah and Burns, 2010), sweet cherry (Smith and Whiting, 2010) and olive (Zahra, 2014). The data herein presented strengthen the direct observation that ethephon aggrandizes both the fruitlet abscission and ethylene evolution in litchi. It is speculated that the mechanism of fruitlet abscission after treatment with exogenous ethephon may relate to the action of ethylene. However, a deep knowledge of the molecular events occurring in FAZ during fruit abscission induced by ethephon is still unknown. Although our previous study cloned a *LcPG1* gene and found its expression in FAZ was paralleled with the alteration of fruitlet abscission in litchi, induced by the ethephon treatment and inhibited by spraying 2,4-dichlorophenoxyacetic acid (2,4-D) (Peng et al., 2013). So this work mainly focused on genome-wide mining putative fruit abscission related genes regulated by ethylene in litchi.

Litchi fruit growth could be divided into two stages (Li et al., 2003). The first Stage constitutes about two thirds of the whole fruit growth cycle, which is the phase mainly characterized by the growth of pericarp and seedcoat; and the second Stage is the phase mainly characterized by the growth of embryo and the rapid aril growth. In the case of “Feizixiao” litchi used in our study, it needs 70–75 days from female flowering to a mature fruit, which can fluctuate some depending on the female opening date (Li et al., 2004). Fruit weight is about 25–30 g at maturity, but it is approximately 1.0 g when fruit develops at 25 days post anthesis (DPA) which is the time of the ETH treatment in this study. There were three to four waves of physiological fruit drop throughout fruit development in 70–90 days depending on cultivars (Yuan and Huang, 1988). Wave I, wave II, and wave III of abscission occurred around 1 week, 3 weeks and 6 weeks after anthesis, respectively. For a normal inflorescence of “Feizixiao” litchi, there are about 500–800 female flowers. Only 10% of them may set fruit successfully at 1 week after anthesis (wave I), and 30–50% of the surviving fruitlet will drop during wave II, after which no abscission will occur for the next 2–3 weeks. This study focuses on fruitlet abscission occurred at the second wave. The first 3 days after treatment might be coincidence with the peak of fruit drop wave II. It is not surprising that ~50% of the control fruitlet abscised between 25 and 28 DPA. Moreover, after 28 DPA, the remaining 50% of the fruitlet in the control treatment did not abscise over the next 2–3 weeks. ETH treatment, however, induced abscission of 100% of the fruitlet by 28 DPA. Thus, the ETH treatment largely magnified the second physiological fruit drop, which inducing a significantly higher rate of fruitlet abscission. This is exactly the biological effect expected for ETH. Moreover, ethylene production in fruit between the control and the ETH treatment had the substantial difference. Ethylene production in the control fruitlet remained more or less flat and kept below 8 μl·kg^−1^·h^−1^ in the period of treatment, while which in ETH-treated fruitlet increased rapidly at 1 d after treatment and achieved nearly a ten-fold higher at 3 d when compared with the control. When the pH is above 4.0, ethephon slowly decomposes to release ethylene. The increase in ethylene production was the results of endogenous synthesis and ethephon release, which probably accelerates the fruit drop. In addition, the CFAR had no difference with the control in the first day after the ETH treatment, after then, the CFAR was sharply increased and significantly higher than that of the control. There were two obvious stages during the fruitlet abscission induced by ethephon: the early induction (0–1 d after treatment) that might induce acquisition of ethylene sensitivity and abscission competence, and the late induction (1–3 d after treatment) that might lead to the execution of fruitlet abscission and formation of the defense layer.

A total of 6167 significantly DEGs were screened as ethylene-responsive genes and 2730 of them were identified as candidate genes involved in the fruitlet abscission process. Over 85% of the candidate genes displayed a significant transient change during the early ethylene-induction. It is generally accepted that ethylene operates as an activator, while auxin act as retardants (Roberts et al., 2002). In agreement with this supposition, 115 out of 195 candidate hormone related genes were involved in biosynthesis and signaling pathway of ethylene and auxin. These evidences were supported by the high expression levels of ethylene signal pathway related genes such as *ETR2*, *EBF*, *EIN3/EIL* and a class of *ERF* TFs, as previously demonstrated by John-Karuppiah and Burns (2010) in sweet orange fruit and leave abscission zone; and the repressed expression of transcript levels for auxin polar transport carriers (*PIN* and *AUX1*) and auxin responsive genes (*TIR1*, *Aux/IAA*, *ARF*, and *SAUR*). A decline in the abundance of auxin efflux carrier might be responsible for fruitlet abscission induced by shading and NAA in apple (Zhu et al., 2011) and mature-fruit abscission in melon (Corbacho et al., 2013). Moreover, Zhu et al. (2011) found that genes involved with cytokinin and gibberellic acid (GA) signaling pathways were down-regulated by shading and NAA in apple fruitlet FAZ. Similarly, a strongly decreased expression of large number of genes related to cytokinin and GA biosynthesis and signaling, such as *CYP735A*, *AHK*, *AHP*, *GA20ox*, and *GID1*, were also found in our study, probably implying that the metabolism of the cytokinin and GA in FAZ were affected in the early ethephon-promoted abscission process. On the other hand, it has been proposed that ABA and JA might be correlated with the abscission activation in citrus fruitlet (Gomez-Cadenas et al., 2000) or leaves (Agustí et al., 2009). They exhibited exactly the opposite results on mature fruit abscission, six of eight DEGs involved in ABA biosynthesis were up-regulated in melon (Corbacho et al., 2013), and six out of the seven DEGs were down-regulated in olive (Gil-Amado and Gomez-Jimenez, 2013). Our result was quite different from them, half of the eight DEGs involved in ABA biosynthesis showed increased transcript abundance during the ethephon-promoted fruitlet abscission in this study. All together, the mentioned above plant hormones were involved in the process of fruitlet abscission induced by the ETH treatment and the most important hormones were ethylene and auxin.

TFs are concerned as major switches of regulatory cascades during development, and the changes in the expression of such genes may affect various biological processes (Riechmann et al., 2000). A total of 185 different TF genes transcript such as *AP2/ERF*, *Aux/IAA*, *bHLH*, *MYB*, *WRKY*, *NAC*, *LBD*, and *HD-ZIP*, were affected by the ETH treatment. Those thought to be directly involved in ethylene and auxin signal transduction, such as *AP2/ERF*, *Aux/IAA*, and *ARF*, were already discussed before. The expression of most genes belonging to the family of *bHLH* was sharply down-regulation, and it was consist with the reports in the flower abscission zone in tomato after flower removal (Meir et al., 2010). Other up-regulated TFs in our work such as *NAC* and *WRKY*, were previously reported to be involved in mature fruit abscission in melon and olive (Corbacho et al., 2013; Gil-Amado and Gomez-Jimenez, 2013). Many reports have shown that numerous characterized *NAC* and *WRKY* genes are involved in response to environmental stimuli and play various roles in response to biotic and abiotic stress (He et al., 2005; Jensen et al., 2010; Zhao et al., 2012). Thus, these up-regulated *NAC* and *WRKY* genes might be similar to ethylene- or stress-induced TFs found in other species (Yang et al., 2009; Jensen et al., 2010) and putatively involved in the downstream of ethylene signaling. Concerning *MYB*, Corbacho et al. (2013) reported that *MYB* was the most represent up-regulated TFs during the late mature-fruit abscission in melon, while only 7 members of the 15 affected *MYB* genes were induced in litchi. All these differential expression of genes encoding TFs belonging to different families might act as early regulators of the abscission induction, but the exact roles of these regulatory factors responding to ethylene induction remain to be further investigated.

ROS are versatile molecules related to a wide range of cellular processes, including programmed cell death, development, and hormonal signaling (Kwak et al., 2006). Previously, some reports supported a link between ROS and abscission. In tobacco, Henry et al. (1974) found that POD activity was increased during the ethylene-induced pedicel abscission. In tomato, delayed abscission of flowers and fruits was related to the increase of ROS-scavenging enzymes (Djanaguiraman et al., 2004). In pepper, Sakamoto et al. (2008) reported that H_2_O_2_ was involved in stress-induced petioles abscission, indicating that H_2_O_2_ acts downstream abscission signaling from ethylene. In ethylene-treated citrus leaves, a set of transcripts belonging to the ROS scavenging machinery have been reported to be over-represented in petioles rather than the laminar abscission zone (Agustí et al., 2008, 2009). Results in this study showed that a number of *Rboh* (role in ROS production) and genes encoding ROS scavenging enzymes were induced, suggesting the burst of ROS caused by ethephon treatment.

Calcium has been considered as an important intracellular messenger in plants and is essential for the maintenance of structural integrity of biomembrane and cell wall (Poovaiah and Rasmussen, 1973), and is also required for a variety of ethylene-dependent abscission processes (Raz and Fluhr, 1992). Xu et al. (2009) reported that direct application of calcium on tomato pedicel explants under ethylene would accelerate abscission but there are a number of reports describing that calcium delayed organ abscission (Poovaiah and Leopold, 1973; Beyer and Quebedeaux, 1974; Iwahori and Van Steveninck, 1988). In fact, Poovaiah and Rasmussen (1973) showed that ethephon treatment for bean leaf explants decreased calcium level in the AZ just prior to separation. The role of this element in organ abscission is still controversial. But how does the calcium signaling communicate with the fruit abscission? Our results showed that ethephon treatment up-regulated genes encoding calcium efflux carrier proteins (*PMCA*), down-regulated different genes encoding calcium influx carrier proteins such as cyclic nucleotide-gated channel (CNGC) genes and calcium responsive genes (*CML* and *CDPK*) in the litchi FAZ-enriched pedicel. These molecular results strongly suggested that regulating calcium influx and maintaining calcium level in the FAZ might be associated with the onset of ethephon-promoted litchi fruitlet abscission process. It is speculated that ethylene might lead to a high extracellular calcium level in FAZ, and result in the deposition of calcium on cell wall and the deficiency on the cell inside.

Ubiquitylation-dependent proteolysis is a major event during both the induction and execution of cell death (Estelle, 2001), and could be triggered by H_2_O_2_ in tobacco (Vandenabeele et al., 2003). In ethylene-treated citrus leaves, a group of transcripts involved in the ubiquitin/proteasome system have been reported to be induced in both laminar abscission zone and petiolar cortical tissue (Agustí et al., 2008, 2009). The up-regulation of both ubiquitin-conjugating enzymes (E2) and ubiquitin-protein ligases (E3) after the ETH treatment in litchi FAZ-enriched pedicel, suggested that a similar proteolytic mechanism might be involved in ethephon-induced abscission. Also, two 26S proteasome components and a large number of proteins with F-box and U-box domain like potential E3 ligases were induced by ethephon. In *Arabidopsis*, abscission of floral organs is arrested with suppressed expression of a F-box protein (González-Carranza et al., 2007). These observations strongly suggested that a general proteasome-related mechanism might play a role in ethephon-induced abscission.

Zhu et al. (2011) found both shading and NAA treatment for apple tree resulted in a large number of photosynthesis-related genes were down-regulated in the FAZ. For instance, 90 out of the 94 DEGs were repressed in shading-treated FAZ. In our study, 103 out of the 110 DEGs involved in photosynthesis pathway were strongly decreased. These results also indicated that photosynthesis was one of the GO terms enriched in FAZ. The structural features of AZ cells were described by Sexton and Roberts (1982) as densely protoplasmic, with small intercellular spaces, containing large deposits of starch, and with a high density of branched plasmodesmata. Then, AZ cells should not contain photosynthetically active chloroplasts. However, hand-dissected AZ-enriched young and green fruitlet pedicle (see Supplementary Figure 1) used in our study should contain photosynthetically active chloroplasts, which is why so many down-regulated of photosynthesis-related genes were found. In order to uncover the abscission-associated metabolism of AZ cells we should take a laser capture microdissection (LCM) approach to obtain AZ- specific cells sample for performing an accurate study of the abscission events in the future. Thus, the roles of these genes on fruit abscission need to be further evaluated.

Some evidences supported a strong connection between the carbohydrate amounts available for the fruit and their probability of abscission (Yuan and Huang, 1988; Iglesias et al., 2003; Zhou et al., 2008). One of the reasons of abscission may be due to a lack of carbohydrate. Not surprisingly, ETH treatment also affected the carbohydrate and energy metabolism. Affected genes within these groups include those associated with glycolysis, TCA cycle and ATPase. These results indicated that ethephon might lead to the carbohydrate stress and the interdiction of ATP synthesis during the early abscission induction. Moreover, an increased ROS production may be linked to the inhibition of ATPase, as a consequence to the mitochondrial damage (Roy et al., 2008), and the release of cytochrome c from mitochondria, which have been implicated as regulator of programmed cell death (Tiwari et al., 2002).

After the early induction, there was a lot of fruitlet dropped in paralleled with a continuous highly release of ethylene yield during the late induction (1–3 d after ETH treatment). Ethylene may act as the signal generated within the fruit, FAZ, or released from ethephon, through diffusion, triggering abscission event. It was supported by the abundant of several transcripts (*ACS*, *ACO*, *ERF*) involved in ethylene biosynthesis and transductive pathway. Several up-regulated genes were linked to ABA biosynthesis and signaling transduction, such as *ZEP* and *PP2C*. It is proposed that ABA might be corrected with the late stage of abscission process. Similarly, ethylene appeared to be a positive regulator of SA action during the abscission induction, since the expression level of *SBP* and *NPR1* genes were highly up-regulated, as found during melon mature-fruit abscission (Corbacho et al., 2013). It assumed that SA might be as an endogenous signaling molecule for stress response or the formation of protective layer after AZ cell separation. Homologs of *LBD* and *WRKY* TFs were also highly expressed at 1–3 d after ETH treatment, showing similar change to that reported in tomato flower abscission zone (Wang et al., 2013). The LBD family played a possible role in lateral meristem initiation (Shuai et al., 2002), while the WRKY family hold central positions mediating the regulation of disease resistance (Robatzek and Somssich, 2001). These TFs might have functions in stress defense and the formation of protective layers after fruitlet abscission. For fruit to be shed, cell separation must occur in FAZ, and the abscission is paralleled with intercellular space increase and middle lamella lysis in the FAZ which is the result of the degradation and/or remodeling of cell wall (Lee et al., 2008; Bowling and Vaughn, 2011). There were 43 cell wall degradation and loosing related transcripts up-regulated and 29 cell wall biosynthesis related genes down-regulated, and most of them had significantly differential expression throughout the whole ethephon treatment, indicating that these genes might be involved in the process of fruitlet abscission induced by ethephon. Several researchers have already reported that the expression of genes encoding for cell wall-hydrolyzing enzymes was associated with abscission and also regulated by ethylene (Lashbrook et al., 1994; Kalaitzis et al., 1997; Roberts and Gonzalez-Carranza, 2009). However, 92 down-regulated genes related to cell wall degradation and loosing, and 15 up-regulated genes related to cell wall biosynthesis were also found in our study, which might be involved in the new cell wall synthesis and reconstruction for the formation of protective layers after fruitlet abscission.

**Figure 7:**
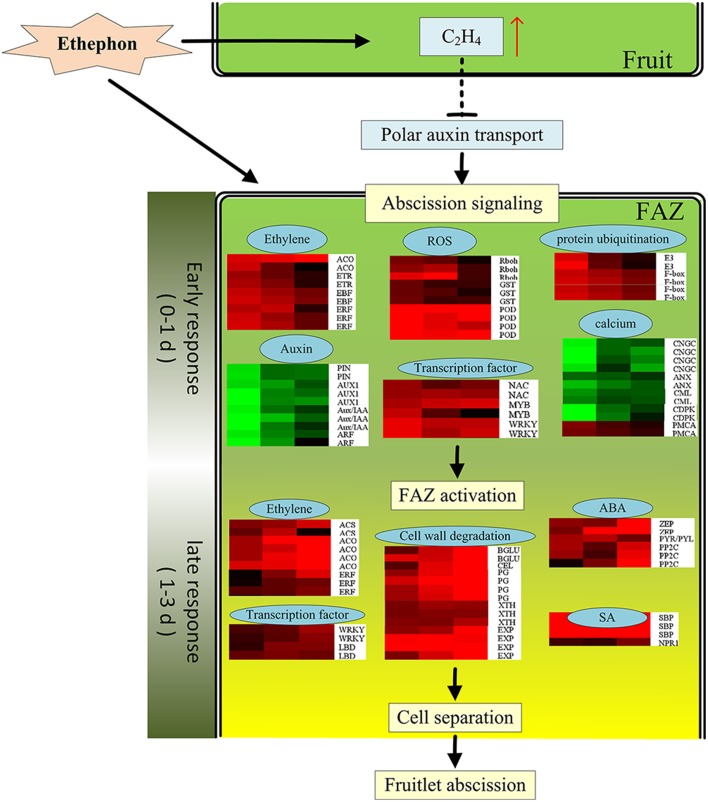
**The possible molecular events to control the ethephon-promoted litchi fruitlet abscission based on expression data obtained from DTA analysis**. Gene expression levels were indicated with color bars: red (up-regulated) and green (down-regulated).

This study only focus on the gene expression profile occurring in the FAZ-enriched pedicel during litchi fruit abscission induced by ethephon. In conclusion, a total of 2730 candidate genes were involved in the process of litchi fruit abscission induced by ethephon treatment. A preliminary molecular regulatory scheme was herein prompted out for litchi fruitlet abscission induced by ethephon based on our results (Figure 7). At the early beginning, the ethylene evolution in fruitlet was greatly increased by ETH treatment, which would suppress the synthesis and polar transport of auxin and trigger abscission signaling. At the same time, FAZ might perceive the abscission signals, and then, 1867 early-responsive genes were up- or down-regulated from 0 to 1 d after ETH treatment. The most affected genes included those related to ethylene biosynthesis and signaling, auxin transport and signaling, TFs, protein ubiquitination, ROS response, calcium signal transduction and etc… Then, a lot of genes related to cell wall degradation, TFs, ethylene, ABA and JA biosynthesis and signaling cascade were up-regulated. At the last, cell separation happened and the fruitlet abscission was enhanced. To the best of our knowledge, this study provides the first global monitoring of gene expression changes occurring in FAZ-enriched pedicel during litchi fruit abscission.

## Author contributions

JL was responsible for the overall concept and experimental design, and revising and finalizing the manuscript. CL carried out ethephon treatment, DTA data integration and analysis, performed qRT-PCR experiments, and drafted the manuscript. YW was responsible for bioinformatics analysis. PY and WM performed the ethephon treatment and sample collection. All the authors read and approved the final manuscript.

### Conflict of interest statement

The authors declare that the research was conducted in the absence of any commercial or financial relationships that could be construed as a potential conflict of interest.

## Supplementary material

Supplementary Figure 1 **The pedicel of a litchi fruitlet showing the pre-existing fruit abscission zone (FAZ) with a visible sunken line (thick arrow)**. The section between the solid lines (~2 mm) was sample for DTA analysis.

Supplementary Figure 2 **Functional enrichment analysis for ethephon-responsive genes. (A)** Enriched GO terms. Hierarchical tree graph of enriched GO terms in agriGO were used to simplify the analysis. The color bar shows the statistical significance, with enrichment significance level *FDR* ≤ 0.05. **(B)** KEGG pathway enrichment analysis. The top 20 enriched pathway are showed. The color bar shows the statistical significance, with enrichment significance level *Q*-value ≤ 0.05. The size of the circle is proportional to the number of genes in the KEGG pathway.

Supplementary Data Excel File 1 **Specific primers details for 41 genes selected for qRT-PCR**. Representative genes selected for the analysis were those involved in plant hormone regulation pathway, cell wall modification, transcription factors regulation, ROS, and calcium signaling. All the gene ID, forward, and reverse primer sequences are included.

Supplementary Data Excel File 2 **List of 6167 differential expressed genes in the three pair-comparisons (CK1 vs. ETH1, CK2 vs. ETH2, and CK3 vs. ETH3)**. We used a false discovery rate (FDR) ≤0.001 and the absolute value of log2 ratio ≥2 as the threshold to judge the significance of transcript abundance differences, and then eliminated the control background influences (CK0 vs. CK1, CK0 vs. CK2, and CK0 vs. CK3).

Supplementary Data Excel File 3 **List of 2730 candidate genes involved in the ethephon-promoted fruitlet abscission process by GO (*FDR* ≤ 0.05) and KEGG (*Q*-value ≤ 0.05) enrichment analysis**.

Supplementary Data Excel File 4 **Plant hormones-related genes induced or repressed in FAZ-enriched pedicel after ethephon treatment**.

Supplementary Data Excel File 5 **Transcription factor genes induced or repressed in FAZ-enriched pedicel after ethephon treatment**.

Supplementary Data Excel File 6 **Cell wall-related genes induced or repressed in FAZ-enriched pedicel after ethephon treatment**.

Supplementary Data Excel File 7 **Photosynthesis, carbohydrate metabolism, and mitochondria-related genes induced or repressed in FAZ-enriched pedicel after ethephon treatment**.

Supplementary Data Excel File 8 **ROS, calcium, and protein ubiquitination-related genes induced or repressed in FAZ-enriched pedicel after ethephon treatment**.
