# Supplementary figures and images for: Genome-wide digital transcript analysis of putative fruitlet abscission related genes regulated by ethephon in litchi
Source: Front Plant Sci. 2015 Jul 7;6:502. doi: 10.3389/fpls.2015.00502 (PMC4493771; doi:10.3389/fpls.2015.00502)

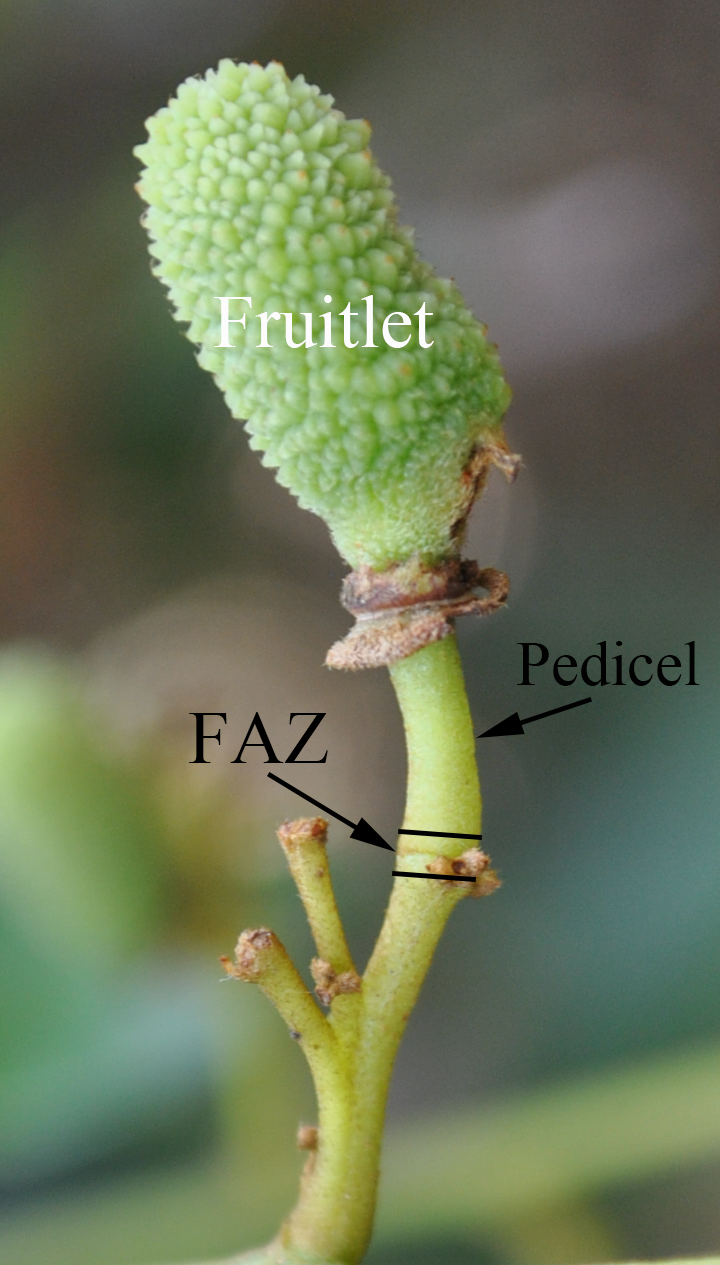

Supplement: Supplementary Figure 1 — The pedicel of a litchi fruitlet showing the pre-existing fruit abscission zone (FAZ) with a visible sunken line (thick arrow). The section between the solid lines (~2 mm) was sample for DTA analysis. [file Image1.TIF]

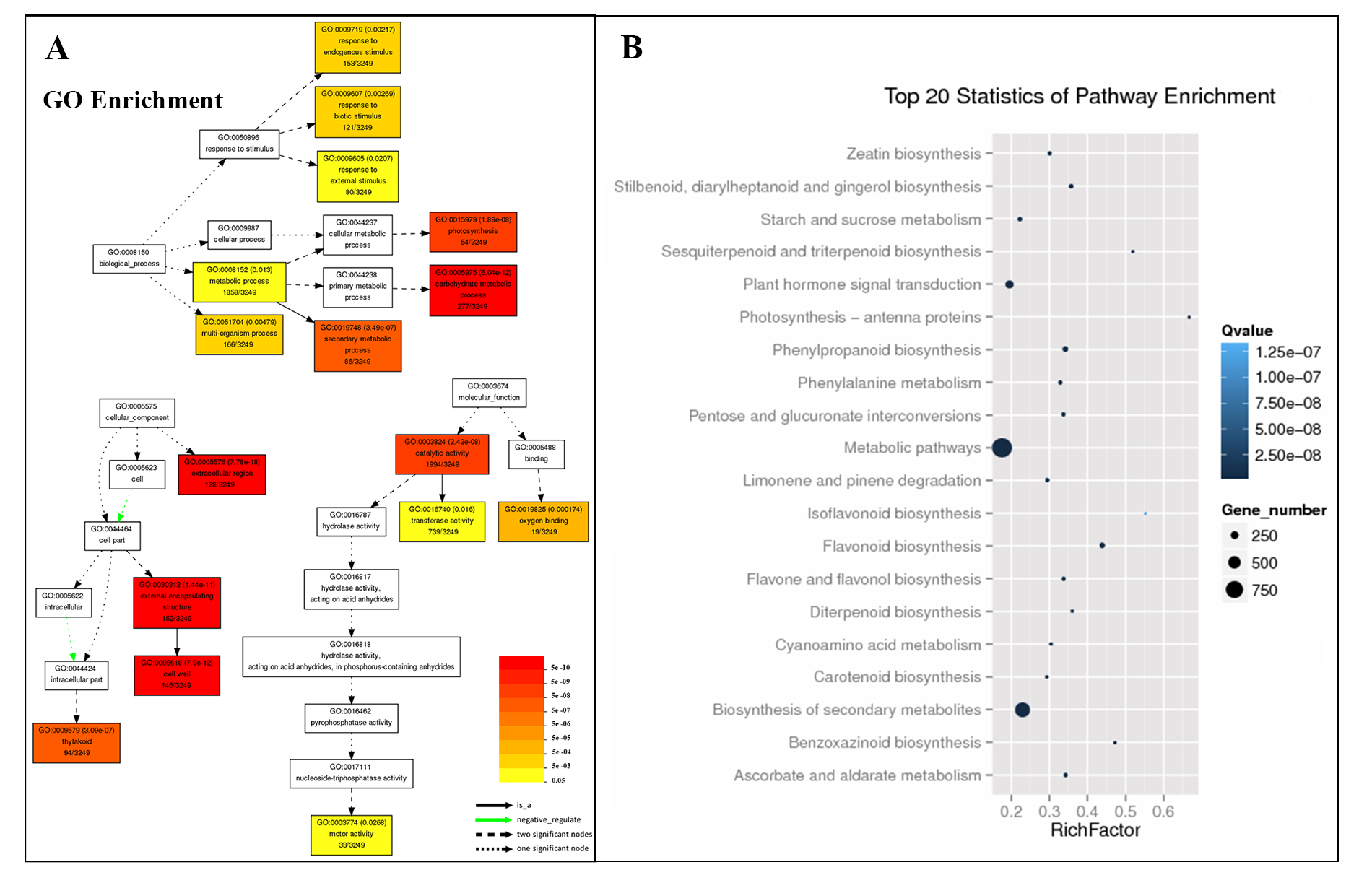

Supplement: Supplementary Figure 2 — Functional enrichment analysis for ethephon-responsive genes. (A) Enriched GO terms. Hierarchical tree graph of enriched GO terms in agriGO were used to simplify the analysis. The color bar shows the statistical significance, with enrichment significance level FDR ≤ 0.05. (B) KEGG pathway enrichment analysis. The top 20 enriched pathway are showed. The color bar shows the statistical significance, with enrichment significance level Q-value ≤ 0.05. The size of the circle is proportional to the number of genes in the KEGG pathway. [file Image2.TIF]
